# Observation of momentum-dependent charge density wave gap in a layered antiferromagnet ${\textrm{Gd}}{\textrm{Te}}_{3}$

**DOI:** 10.1038/s41598-023-44851-8

**Published:** 2023-10-30

**Authors:** Sabin Regmi, Iftakhar Bin Elius, Anup Pradhan Sakhya, Dylan Jeff, Milo Sprague, Mazharul Islam Mondal, Damani Jarrett, Nathan Valadez, Alexis Agosto, Tetiana Romanova, Jiun-Haw Chu, Saiful I. Khondaker, Andrzej Ptok, Dariusz Kaczorowski, Madhab Neupane

**Affiliations:** 1 Department of Physics, University of Central Florida, Orlando, FL 32816 USA; 2 NanoScience Technology Center, University of Central Florida, Orlando, FL 32826 USA; 3 Institute of Low Temperature and Structure Research, Polish Academy of Sciences, Okólna 2, 50-422 Wrocław, Poland; 4 Department of Physics, University of Washington, Seattle, WA 98195 USA; 5 Institute of Nuclear Physics, Polish Academy of Sciences, W. E. Radzikowskiego 152, 31342 Kraków, Poland; 6 Present Address: Center for Quantum Actinide Science and Technology, Idaho National laboratory, Idaho Falls, ID 83415 USA

**Keywords:** Materials science, Physics

## Abstract

Charge density wave (CDW) ordering has been an important topic of study for a long time owing to its connection with other exotic phases such as superconductivity and magnetism. The $R{\textrm{Te}}_{3}$ (*R* = rare-earth elements) family of materials provides a fertile ground to study the dynamics of CDW in van der Waals layered materials, and the presence of magnetism in these materials allows to explore the interplay among CDW and long range magnetic ordering. Here, we have carried out a high-resolution angle-resolved photoemission spectroscopy (ARPES) study of a CDW material ${\textrm{Gd}}{\textrm{Te}}_{3}$, which is antiferromagnetic below $\sim \mathrm {12~K}$, along with thermodynamic, electrical transport, magnetic, and Raman measurements. Our ARPES data show a two-fold symmetric Fermi surface with both gapped and ungapped regions indicative of the partial nesting. The gap is momentum dependent, maximum along ${\overline{\Gamma }}-\mathrm{\overline{Z}}$ and gradually decreases going towards ${\overline{\Gamma }}-\mathrm{\overline{X}}$. Our study provides a platform to study the dynamics of CDW and its interaction with other physical orders in two- and three-dimensions.

## Introduction

Charge density wave (CDW)^1,2^ in quantum materials have been a subject of numerous research works for a number of decades because of its relevance in understanding several physical properties and also its competition or coexistence with exotic phases like superconductivity and magnetism^3–14^. CDW ordering is a phenomenon associated with Fermi surface (FS) instabilities, where a periodic lattice distortion leads to the spatial modulation of carrier density^2^. One example is the Peierls distortion in one-dimension^15,16^, in which the lattice periodicity can be doubled by electronically disturbing the system with a wave vector that is double the Fermi wave number, resulting into a gap opening at the Brillouin zone (BZ) boundaries nested by the same wave vector (FS nesting). With increase in dimensions, the FS nesting tends to be imperfect so that certain regions of the FS remain ungapped, leading to a metallic nature^17,18^. The mechanism of CDW in such higher-dimensional materials is still of great interest as it can differ from material to material, can have different origin, and also depends on crystal growth conditions^19–22^.

The orthorhombic crystalline family of van der Waals layered materials $R{\textrm{Te}}_{3}~(R~=~\mathrm {rare-earth~elements})$ has been broadly studied for the presence of CDW^23–27^. The CDW ordering takes place at a high temperature, and materials with heavier rare-earth elements exhibit a second CDW transition at a lower temperature^28,29^. In addition, the existence of long range magnetic ordering in these compounds provides ground to study the interplay among magnetic and CDW orders^30,31^. $R-\textrm{Te}$ slabs are sandwiched in between planar bi-layers of $\textrm{Te}-\textrm{Te}$, where the neighboring $\textrm{Te}-\textrm{Te}$ are connected through weak van der Waals interaction, thereby easing the exfoliation of these layered materials to the two-dimensional limit^32–35^. Angle-resolved photoemission spectroscopy (ARPES)^36,37^ has been a useful tool to directly probe the energy-momentum dispersion in quantum materials. It has been extensively used to study the electronic structure of $R{\textrm{Te}}_{3}$ in the investigation of FS and CDW induced gap^17,38–47^. The gap size is found to depend on momentum, and the maximum gap changes as a function of the rare-earth element *R*, which can be modeled by a nesting driven sinusoidal CDW^40^. Among the materials under this family, ${\textrm{Gd}}{\textrm{Te}}_{3}$ has been reported to have very high electronic mobility^33^ and steep band dispersion at the Fermi level^45^. It can be thinned down to ultrathin limit using mechanical exfoliation that allows to study the thickness dependence of the CDW ordering^32,33^. Properties such as CDW ordering, magnetic ordering, and pressure induced superconductivity^28,31,48^ make this system interesting in order to explore more on the electronic properties.

**Figure 1:**
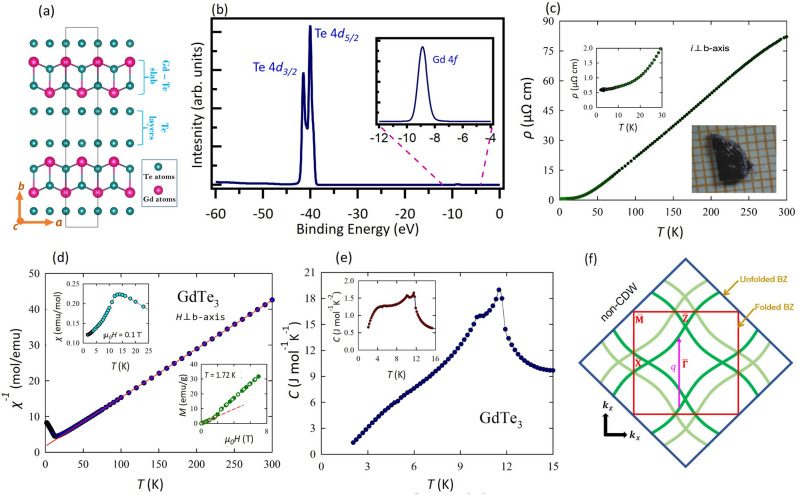
Crystal structure and sample characterization of ${\textrm{Gd}}{\textrm{Te}}_{3}$. (**a**) Crystal structure of ${\textrm{Gd}}{\textrm{Te}}_{3}$, where the pink and the teal colored balls represent $\textrm{Gd}$ and $\textrm{Te}$ atoms, respectively. (**b**) Spectroscopic core level spectrum showing peaks of $\textrm{Gd}~4f$ and $\textrm{Te}~4d$ peaks. (**c**) Temperature dependence of the electrical resistivity measured with electrical current flowing in the crystallographic $a-c$ plane. Inset: the low-temperature resistivity data. (**d**) Temperature variation of the inverse magnetic susceptibility measured in a magnetic field of $\mathrm {0.1~T}$ applied perpendicular to the crystallographic *b*-axis. Solid line represents the Curie-Weiss fit described in the text. Top left inset: The low-temperature magnetic susceptibility data. Bottom right inset: Magnetic field variation of the magnetization taken at $\mathrm {1.72~K}$ with increasing (full circles) and decreasing (open circles) field strength. Dashed line emphasizes a linear behavior in small fields and the metamagnetic transition near $\mathrm {1.5~T}$. (**e**) Low-temperature dependence of specific heat near the magnetic transition. Inset: Same data plotted as the ratio of specific heat to temperature versus temperature. (**f**) Schematic of non-CDW FS in $R\textrm{Te}_3$ showing two-dimensional BZ of $\textrm{Te}$ plane and three-dimensional BZ from the three-dimensional unit cell.

**Figure 2:**
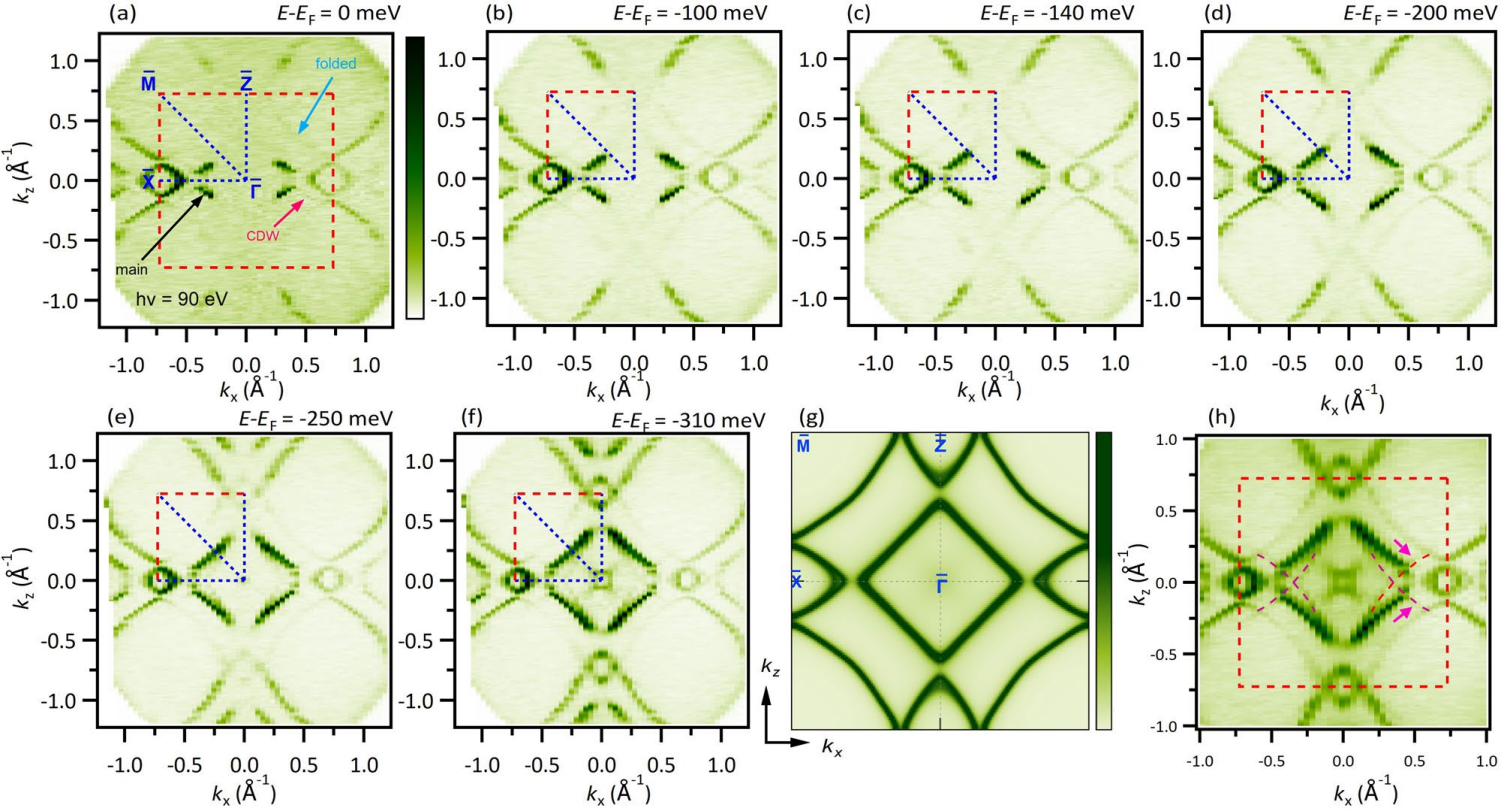
Constant energy contours on the (010) surface of ${\textrm{Gd}}{\textrm{Te}}_{3}$. (**a**) ARPES measured FS with a photon energy of $\mathrm {90~eV}$. Experimental BZ is shown with red dashed lines. High-symmetry points and directions are labeled in blue color. (**b–f**) Energy contours at various binding energies as noted on top of each plot. (**g**) Calculated FS for non-CDW case. (**h**) Energy contour integrated within $\mathrm {50~meV }$ window centered at $\mathrm {300~meV}$ binding energy. ARPES data were collected at the SSRL end-station 5-2 at a temperature of $\mathrm {8~K}$.

In this communication, by utilizing high-resolution ARPES measurements, we study the electronic structure of layered van der Waals material ${\textrm{Gd}}{\textrm{Te}}_{3}$. Our thermodynamic and electrical transport measurements show that the material is antiferromagnetic (AFM), with the magnetic transition occurring below $\sim \mathrm {12~K}$. The CDW transition occurs well above room temperature, at around $\sim \mathrm {375~K}$. Our ARPES results show two-fold symmetric FS with spectral intensity absent around certain regions of the FS, especially around ${\overline{\Gamma}}-\mathrm{\overline{M}}$ to ${\overline{\Gamma}}-\mathrm{\overline{Z}}$, indicating the presence of gap at the Fermi level. Some regions of the FS near the $\mathrm {\overline{X}}$ point remain gapless, implying partial nesting. This gap is strongly direction dependent, with a gap maximum along ${\overline{\Gamma}}-\mathrm{\overline{Z}}$ and gradually decreasing towards ${\overline{\Gamma}}-\mathrm{\overline{X}}$. The room temperature Raman spectroscopy measurement shows the presence of CDW amplitude mode in the bulk as well as ultrathin samples up to 4L ($\sim \mathrm {5~nm}$). Our results indicate that this material is excellent for studying the dynamics and interaction of CDW with other physical parameters in both three- and two-dimensional limits.

## Results

### Crystal structure and bulk properties measurements

${\textrm{Gd}}{\textrm{Te}}_{3}$ crystallizes in a layered orthorhombic structure (space group Number 63) with lattice parameters $a \approx c \approx \mathrm {4.33~{\text{\AA }}}$ and $b = \mathrm {25.28~\text{\AA }}$, close to the values reported in literature^33^. In Fig. 1a, we present the side view of the crystal structure of ${\textrm{Gd}}{\textrm{Te}}_{3}$, where pink balls represent the gadolinium atoms and teal colored balls represent the tellurium atoms. The crystal structure is composed of $\mathrm {Gd - Te}$ slabs sandwiched in between the $\textrm{Te}$ bi-layers. Neighboring $\textrm{Te}$ layers are bonded by weak van der Waals interaction, and the natural cleaving plane is parallel to the *ac* plane—the (010) plane. In Fig. 1b, we show the spectroscopic core level spectrum, where peaks associated with $\textrm{Te}$ 4*d* and $\textrm{Gd}$ 4*f* can be clearly identified.

${\textrm{Gd}}{\textrm{Te}}_{3}$ exhibits a CDW phase transition, with the transition temperature well above room temperature ($\sim \mathrm {377~K}$)^28^. In addition, it also undergoes an AFM transition at a low temperature of $\sim \mathrm {12~K}$^31,33,49^. Our heat capacity (*C*) measurements also show an anomaly near $\mathrm {375~K}$ (see Supplementary Note 1 & Supplementary Fig. 1] that arises due to the incommensurate charge density wave formation^27^. The room temperature Raman spectrum for the bulk crystal shows a CDW amplitude mode along with other phonon modes, in concert with the literature^32,51^, establishing the presence of room-temperature CDW in the material. The mode remains prominent when the bulk crystal is thinned down to 4-layered samples via gold-assisted mechanical exfoliation, which suggests this material to be an excellent platform to study the interplay of CDW and long-range orders down to the two-dimensional limit as well [see Supplementary Note 2 & Supplementary Fig. 2]. Figure 1c displays the temperature dependence of the electrical resistivity ($\rho$) of ${\textrm{Gd}}{\textrm{Te}}_{3}$ measured with electrical current flowing in the crystallographic *ac*-plane. In concert with the previous reports^28,31,33^, single crystals of ${\textrm{Gd}}{\textrm{Te}}_{3}$ investigated in the present study exhibit very good metallic-type charge conductance. The ratio of the resistivity values measured at $\mathrm {300~K}$ and $\mathrm {2~K}$ is as large as $\textrm{140}$, thus indicating high crystalline quality of the samples. On approaching the room temperature, the $\rho (T)$ curve clearly changes its slope signaling the proximity of the CDW transition. Figure 1d displays the temperature dependence of the inverse magnetic susceptibility ($\chi ^{-1}$) measured for magnetic field applied perpendicular to the crystallographic *b*-axis. Above about $\mathrm {30~K}$, $\chi ^{-1}(T)$ exhibits a straight line behavior that can be approximated by the Curie-Weiss formula with the effective magnetic moment $\mu _{eff} = \mathrm {7.67~\mu _B}$ and the paramagnetic Curie temperature $\theta _p = \mathrm {-12.6~K}$. The value of $\mu _{eff}$ is close to the theoretical prediction for $\textrm{Gd}^{3+}$ ion. The negative value of $\theta _p$ reflects the predominance of AFM exchange interactions, which bring about the long range AFM ordering below about $\mathrm {12~K}$ (see the upper inset to Fig. 1d). The AFM nature of the electronic ground state in ${\textrm{Gd}}{\textrm{Te}}_{3}$ is further corroborated by the characteristic behavior of the magnetization isotherm taken at $T = \mathrm {1.72~K}$ (see the lower inset in Fig. 1d) with a clear metamagnetic transition near $\mathrm {1.5~T}$. Overall, the magnetic data collected in our study agrees very well with those reported in the literature^30,33,49^. We also observed a distinct lambda-shaped anomaly at around $\mathrm {11.6~K}$ in *C*(*T*) graph, followed by a subsequent feature observed at $\mathrm {10~K}$ (Fig. 1e), similar to previous reports^31,33,49^.

**Figure 3:**
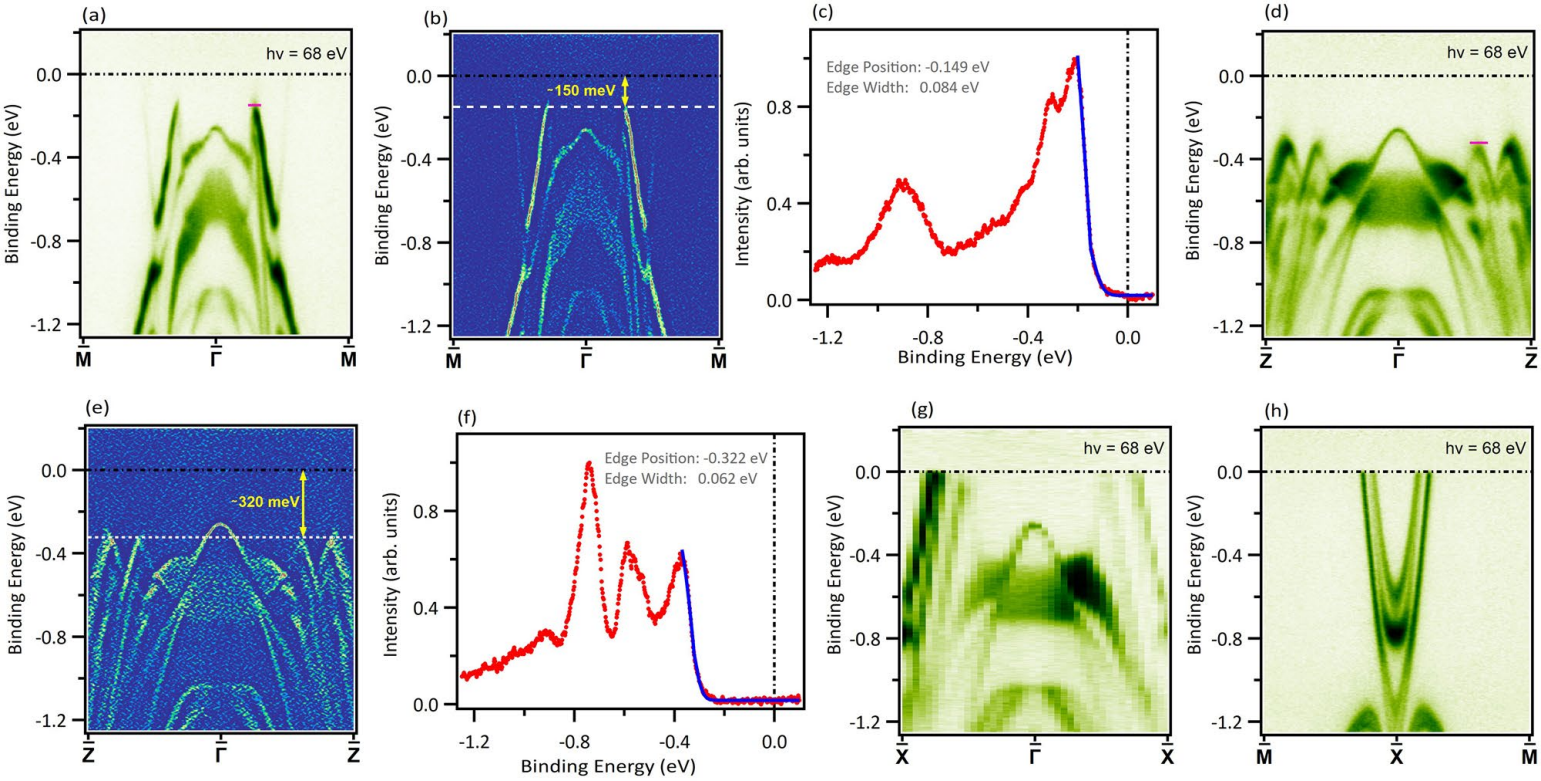
Experimental observation of CDW induced gap. (**a**) Dispersion map and (**b**) its second derivative along the $\mathrm{\overline{M}}-{\overline{\Gamma}}-\mathrm{\overline{M}}$ direction. (**c**) Energy distribution curve integrated within the momentum window represented by magenta line in (**a**) and the Fermi edge fit. (**d**) Dispersion map and (**e**) its second derivative along the $\mathrm{\overline{Z}}-{\overline{\Gamma}}-\mathrm{\overline{Z}}$ direction. (**f**) Energy distribution curve integrated within the momentum window represented by the magenta line in (**d**) and the Fermi edge fit. (**g**) Experimental dispersion map along the $\mathrm{\overline{X}}-{\overline{\Gamma}}-\mathrm{\overline{X}}$ direction. (**h**) Experimental band structure along $\mathrm{\overline{M}}-\mathrm{\overline{X}}-\mathrm{\overline{M}}$. Data were collected at the SSRL beamline 5-2 at a temperature of $\mathrm {8~K}$.

### Experimental observation of the CDW gap

In Fig. 1e, we present a schematic of the non-CDW FS of $R\textrm{Te}_3$. The bands crossing the Fermi level come from the *p* orbitals of the atoms within the $\textrm{Te}$ layers. The two-dimensional unfolded band structure (dark green bands referred to as main bands hereafter) corresponds to ”true” square lattice with one $\textrm{Te}$ atom in the primitive unit cell. Such unit cell can be obtained by $\sqrt{2}$ times reduction and ${45}^{\circ }$ rotation of the unit cell of the whole crystal structure. To account for the three-dimensional lattice symmetry, the FS is acquired by considering the folding of the bands in the $\textrm{Te}$ plane leading to the folded BZ represented by the red square in Fig. 1e. The band folding is reflected on the observed band structure, where the mismatch between bands lead to their occurrence as the low intensity shadow bands ^40,42^. In Fig. 1e, $q = \frac{5}{7}c^*$ represents a nesting condition, where the wave vector nests two sets of the main bands, which would still be present if we only consider the two-dimensional unit cell of the $\textrm{Te}$ plane without folding.

The constant energy contours obtained at the FS and at various binding energies using a photon source of energy $\mathrm {90~eV}$ ($T = \mathrm {8~K}$) are presented in Fig. 2. The BZ represented by the dashed red lines is obtained from the (010) surface projection of the three-dimensional BZ. As seen in Fig. 2a, the FS is metallic, in agreement with the metallic nature observed in the transport measurements, and is two-fold symmetric. Strong photoemission intensity is observed at the Fermi level along and around the ${\overline{\Gamma}}-\mathrm{\overline{X}}$ direction. However, the intensity for the main bands along the ${\overline{\Gamma}}-\mathrm{\overline{M}}$ and ${\overline{\Gamma}}-\mathrm{\overline{Z}}$ directions is missing. On going to lower binding energy of about $\mathrm {100~meV}$, the intensity of the main bands starts to fill up towards the ${\overline{\Gamma}}-\mathrm{\overline{M}}$ line, however, a gap still exists along the ${\overline{\Gamma}}-\mathrm{\overline{M}}$ and ${\overline{\Gamma}}-\mathrm{\overline{Z}}$ directions. The main band intensity only fills up along the ${\overline{\Gamma}}-\mathrm{\overline{M}}$ line at around $\sim \mathrm {140~meV}$ binding energy and along the ${\overline{\Gamma}}-\mathrm{\overline{Z}}$ line at $\sim \mathrm {310~meV}$ binding energy. These results indicate that a gap exists at the Fermi level along the ${\overline{\Gamma}}-\mathrm{\overline{M}}$ and ${\overline{\Gamma}}-\mathrm{\overline{Z}}$ directions. Similar nature of the gap was obtained in different set of measurements performed using $\mathrm {68~eV}$ incident photon energy, in which the main band intensity appears along the ${\overline{\Gamma}}-\mathrm{\overline{M}}$ and ${\overline{\Gamma}}-\mathrm{\overline{Z}}$ directions, only around the binding energies of $\sim \mathrm {150~meV}$ and $\sim \mathrm {320~meV}$ binding energies, respectively [see Supplementary Note 3 & Supplementary Fig. 3]. In Fig. 2h, we present the theoretically calculated FS (without considering CDW), which is similar to the one presented in Fig. 1e. To compare with the theoretical FS, we present the experimental energy contour in Fig. 1e, which is integrated up to $\sim \mathrm {300~meV}$ binding energy. Main bands as well as the shadow bands (from the folding), as previously described, can be observed. In addition to the low intensity bands coming from the folding of the three-dimensional BZ, we also observe other faint bands (shown by magenta colored arrows and traced by magenta colored curves) that are not captured in the calculations [Also see Supplementary Note 4 and Supplementary Fig. 4]. These bands arise as a result of the CDW ordering.

In order to quantify the gap along the ${\overline{\Gamma}}-\mathrm{\overline{M}}$ and ${\overline{\Gamma}}-\mathrm{\overline{Z}}$ directions, we took cuts along these directions and analyzed the corresponding band dispersion. In Fig. 3a, we present the dispersion map along ${\overline{\Gamma}}-\mathrm{\overline{M}}$ obtained using a photon source of $\mathrm {68~eV}$ at a temperature of $\mathrm {8~K}$. From the band dispersion, there exists a gap along this direction line with the absence of photoemission signal in the FS. The second derivative plot of the band dispersion presented in Fig. 3b shows that the bands extend up to the binding energy of about $\sim \mathrm {150~meV}$, and a clear gap exists above this binding energy along the ${\overline{\Gamma}}-\mathrm{\overline{M}}$ direction. The gap of about $\mathrm {150~meV}$ below the Fermi level can also be seen from the Fermi fit of the leading edge in the energy distribution curve (Fig. 3c) taken within the momentum window represented by the magenta colored solid line in Fig. 3a. Next, we turn our attention to explore the gap along ${\overline{\Gamma}}-\mathrm{\overline{Z}}$. From the dispersion map (Fig. 3d), its second derivative (Fig. 3e), and the Fermi fit of the leading edge in energy distribution curve (Fig. 3f), it is clear that a gap below the Fermi level of about $\sim \mathrm {320~meV}$ exists along this direction. The dispersion maps for $\mathrm {90~eV}$ incident photon energy are presented in the Supplementary Fig. 5 [also see Supplementary Note 5], which show that gaps along the ${\overline{\Gamma}}-\mathrm{\overline{M}}$ and ${\overline{\Gamma}}-\mathrm{\overline{Z}}$ directions are $\sim \mathrm {140~meV}$ and $\sim \mathrm {310~meV}$, respectively, as observed in the energy contours presented in Fig. 2. The calculated band structures along these directions well reproduce the experimental data barring the CDW induced gap as the calculations are carried out for the non-CDW case. ARPES can only probe up to the Fermi energy, so the value of the total gap size can not be obtained from the ARPES measurements. Along ${\overline{\Gamma}}-\mathrm{\overline{X}}$ and $\mathrm{\overline{M}}-\mathrm{\overline{X}}$, however, bands cross the Fermi level [Fig. 3g,h; also see Supplementary Note 6 & supplementary Fig. 6], which is in accordance with the photoemission intensity observed along this direction in the energy contours in Fig. 2.

### Momentum dependence of the CDW gap below the Fermi level

**Figure 4:**
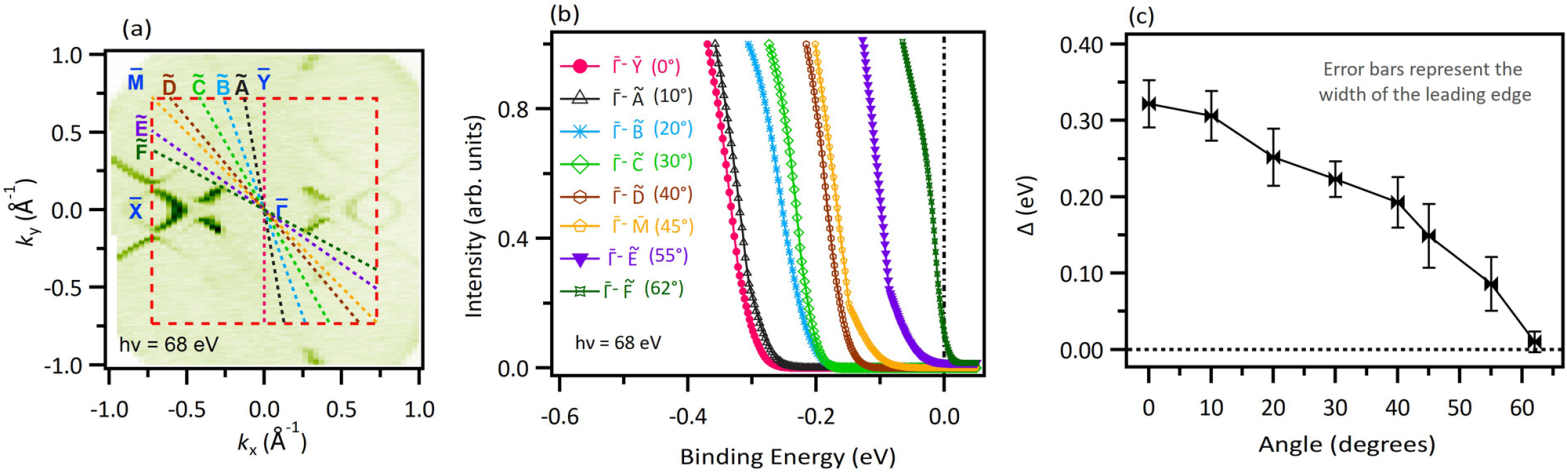
Momentum dependence of the gap. (**a**) FS measured with photon energy of $\mathrm {68~eV}$ with colored lines representing the cuts at color-coded angles. The angles are taken with respect to the $\mathrm{\overline{Z}}-{\overline{\Gamma}}-\mathrm{\overline{Z}}$ direction. (**b**) Shift in the fitted Fermi edge on going away from $\mathrm{\overline{Z}}-{\overline{\Gamma}}-\mathrm{\overline{Z}}$. (**c**) Gap below the Fermi level plotted against the angle from the $\mathrm{\overline{Z}}-{\overline{\Gamma}}-\mathrm{\overline{Z}}$ direction. Data were collected at the SSRL beamline 5-2 at a temperature of $\mathrm {8~K}$.

From the observations in Figs. 2 and 3, we get the idea that the spectral intensity corresponding to the main bands appears at lower and lower binding energies as we move from ${\overline{\Gamma}}-\mathrm{\overline{X}}$ to ${\overline{\Gamma}}-\mathrm{\overline{Z}}$. In Fig. 4, we analyze this momentum dependence of the gap below the Fermi level in measurements using a photon energy of $\mathrm {68~eV}$ as a function of counter-clockwise angle from the ${\overline{\Gamma}}-\mathrm{\overline{Z}}$ direction. $\mathrm {0^{\circ }}$ corresponds to the ${\overline{\Gamma}}-\mathrm{\overline{Z}}$ direction and a $\mathrm {45^{\circ }}$ counter-clockwise rotation means we are looking at the ${\overline{\Gamma}}-\mathrm{\overline{M}}$ direction. Figure 4b represents the leading edges in the integrated energy distribution curves for dispersion maps corresponding to different angles defined in Fig. 4a. It is clear that the leading edge shifts towards the Fermi level when we take the dispersion map away from the ${\overline{\Gamma}}-\mathrm{\overline{Z}}$ direction towards the ${\overline{\Gamma}}-\mathrm{\overline{X}}$ direction, indicative of reducing gap below the Fermi level. At an angle of $\mathrm {62~^{\circ }}$, the gap size surpasses the limits of the experimental energy resolution, therefore, it can be anticipated that the gap vanishes at greater angles [also see Supplementary Note 7 & Supplementary Fig. 7]. In Fig. 4c, we plot the gap below the Fermi level as a function of the counter-clockwise angle from ${\overline{\Gamma}}-\mathrm{\overline{Z}}$, where with angle, the gap gradually reduces from around $\mathrm {320~meV}$ at $\mathrm {0^{\circ }}$ to around $\mathrm {150~meV}$ at $\mathrm {45^{\circ }}$, i.e., ${\overline{\Gamma}}-\mathrm{\overline{M}}$ direction and reaches out of experimental resolution at angles greater than $\mathrm {62^{\circ }}$.

## Discussion

Given the existence of CDW ordering and AFM ordering in $$R\textrm{Te}_3$$ materials, in which 4*f* electrons coming from the *R*-element may bring electronic correlations into play, these materials are excellent platforms to study the interplay among electronic interactions, CDW, and long-range magnetic orders. The layered nature of the crystal structure with very weak van der Waals interaction provides opportunity to study such interplay in both three- and two-dimensions as mechanically exfoliating the crystals to the two-dimensional limit is easy. In this study, we studied bulk crystals of one of such materials $$\textrm{Gd}\textrm{Te}_3$$, which exhibits CDW transition at $$\sim \mathrm {375~K}$$ and the magnetic ordering onsets below $$\sim \mathrm {12~K}$$^10,28,31,33,49^. The $$\textrm{Gd}~4f$$ states are well below the Fermi level as seen in the spectroscopic core level measurement. We were able to obtain thin flakes of $$\textrm{Gd}\textrm{Te}_3$$ via mechanical exfoliation, which still showed prominent CDW amplitude peak in the Raman spectrum taken at room temperature, indicating the presence of high-temperature CDW in the ultrathin limit as well. In fact, the CDW transition temperature is expected to increase with lowering of the sample thickness^32^. The FS obtained from ARPES measurement is two-fold symmetric. This two-fold symmetry instead of four-fold symmetry is as a result of slightly greater *c* than *a* ($$c-a\sim \mathrm {0.01~\text{\AA }}$$^33^), and this changes possible two degenerate CDWs along *a* and *c* axes in favor of a single CDW along *c* direction with a CDW wave-vector $$q_{CDW} = \frac{2}{7}c^*$$^23,26,28^. This wave vector connects the inner and outer diamond sheets in the FS, formed by the unfolded and folded band structures, respectively. An equivalent wave vector $$q = c^*-q_{CDW} = \frac{5}{7}c^*$$ nests the FS made by the main bands coming from a ”true” two-dimensional plane of $$\textrm{Te}$$ atoms and therefore would be present even if the effects of the band folding scenario are not considered^28,38,50^. In our experimental data, the FS features coming from the shadow folded bands seem to be present even at the FS, however, the main band intensity is only along and around the $${\overline{\Gamma}}-\mathrm{\overline{X}}$$ direction and absent going away from this direction around $${\overline{\Gamma}}-\mathrm{\overline{M}}$$ and $${\overline{\Gamma}}-\mathrm{\overline{Z}}$$ directions. Therefore, our study shows that the CDW gap in $$\textrm{Gd}\textrm{Te}_3$$ is governed by the nesting condition $$q = \frac{5}{7}b^{*}$$. Probing of the CDW gap is restricted to below the Fermi level, as the ARPES spectral function depends upon Fermi-Dirac function, which is zero above the Fermi energy. The gap below the Fermi level is strongly dependent on the direction along which we take the dispersion map - it is highest along $${\overline{\Gamma}}-\mathrm{\overline{Z}}$$ and reduces gradually towards $${\overline{\Gamma}}-\mathrm{\overline{X}}$$, vanishing before reaching $${\overline{\Gamma}}-\mathrm{\overline{X}}$$ line. Such a nature of the gap occurs in nesting driven CDW when the nesting is imperfect and has been reported in a previous study on $$R{\textrm{Te}}_{3}$$ which reports the nesting to become imperfect away from $$k_x = 0$$^40^. Measurement performed at a different temperature of $$\mathrm {15~K}$$ (conducted at a different beamline setup) reveals similar dependence on momentum (see Supplementary Fig. 8). In addition to the CDW induced gap, we are able to identify some features that can not be described in the non-CDW FS with main and folded bands. These features also appear as shadow-like bands with weak intensity and are expected to originate due to the reconstruction caused by the CDW ordering.

To conclude, we studied a van der Waals layered material, ${\textrm{Gd}}{\textrm{Te}}_{3}$, by performing high-resolution ARPES measurements of the electronic structure. We were able to probe the CDW induced gap as well as band features originating from the CDW ordering-induced reconstruction. The gap obtained in our study is strongly dependent on the momentum direction with the highest gap lying along the ${\overline{\Gamma}}-\mathrm{\overline{Z}}$ direction. A prominent peak associated with the CDW amplitude mode is seen in our Raman measurements in samples as thin as 4L. Overall, our study demonstrates that ${\textrm{Gd}}{\textrm{Te}}_{3}$ is an excellent material platform to investigate the dynamics of CDW and its connection with other physical orders like magnetism and superconductivity as a function of sample thickness ranging from three- to two-dimensions.

## Methods

### Crystal growth and characterization

High-quality single crystals of $\textrm{Gd}\textrm{Te}_3$ were synthesized using the self-flux technique, as described in the literature^52^. Chemical composition and phase homogeneity of the crystals were determined by means of energy-dispersive x-ray (EDX) analysis performed using a FEI scanning electron microscope equipped with an EDAX Genesis XM4 spectrometer. The specimens examined were found homogeneous and single-phase. The crystal structure was verified by powder x-ray diffraction (XRD) made on finely pulverized crystals employing a PANanalytical X’pert Pro diffractometer with $\mathrm {Cu~K_\alpha }$ radiation. The XRD data was evaluated using the Rietveld method and the FULLPROF software package. The result confirmed the orthorhombic structure (space group *#63*) and yielded the lattice parameters $a \approx c \approx \mathrm {4.33~\text{\AA }}$, $b = \mathrm {25.3~\text{\AA }}$, close to values reported in literature^33^. In addition, the single crystal selected for physical properties measurements was examined on an Oxford Diffraction X’calibur four-circle single-crystal x-ray diffractometer equipped with a CCD Atlas detector.

### Thermodynamic and electrical transport measurements

Magnetic properties were investigated from $\mathrm {1.72~K}$ to $\mathrm {300~K}$ and in magnetic fields up to $\mathrm {7~T}$, applied perpendicular to the crystallographic *b*-axis, using a Quantum Design MPMS-XL superconducting quantum interference device (SQUID) magnetometer. The heat capacity was measured in the temperature interval $\mathrm {2-400~K}$ employing the relaxation technique and two-$\tau$ model implemented in a Quantum Design PPMS-9 platform.

Electrical transport measurements were performed on a bar-shaped specimen cut from the oriented crystal using a wire saw. Electrical contacts were made by silver wires of diameter $\mathrm {20~\mu m}$, attached to the specimen’s surface in a linear manner with a single-component silver paste. The experiments were carried out in the same PPMS platform in the temperature range $\mathrm {2-300~K}$ employing a standard four-points ac technique and electrical current flowing within the crystallographic *ac*-plane.

### Raman spectroscopy measurements

Raman spectroscopy measurements were performed in ambient conditions using a Horiba LabRAM HR Evolution Spectrometer equipped with a $\mathrm {1800~grooves/mm}$ grating and a Synapse EMCCD detector. A frequency doubled Nd:YAG $\mathrm {532~nm}$ excitation laser source was focused to a square micron sized beam spot using an $\mathrm {100\times }$ objective. An ultra-low-frequency (ULF) filter was utilized to resolve the CDW peak at low wavenumbers.

### Spectroscopic characterization

High-resolution ARPES measurements were performed at the Stanford Synchrotron Radiation Lightsource (SSRL) end-station 5-2 and at the Advanced Light Source (ALS) beamline 10.0.1.2, both equipped with a DA30 electron analyzer. The angular and energy resolutions were set better than $\mathrm {0.2^{\circ }}$ and $\mathrm {20~meV}$, respectively. The samples, mounted on copper posts and attached to ceramic posts on top using silver epoxy paste, were loaded and cleaved *in situ* inside the ARPES chamber maintained under ultra high vacuum better than $\mathrm {10^{-10}~torr}$. Measurements at SSRL were carried out at a temperature of $\mathrm {8~K}$ and those at ALS were performed at $\mathrm {15~K}$.

### Computational details

The first-principles calculations based on density-functional theory (DFT)^53,54^ were performed using the projector augmented-wave (PAW) potentials ^55^ implemented in the QuantumESPRESSO^56–58^. The calculations were performed within the generalized gradient approximation (GGA) in the Perdew, Burke, and Ernzerhof (PBE) parameterization^59^, develop within PsLibrary^60^.

The atom position was optimized for conventional unit cell with experimental values of lattice vectors, using the $\textrm{14} \times \textrm{2} \times \textrm{14}$ **k**–point grid in the Monkhorst–Pack scheme ^61^. As the convergence condition for the optimization loop, we took the energy difference of $\mathrm {10^{-6}~eV}$. The calculations were performed with the energy cut-off set to $\mathrm {400~eV}$. In calculations, the $\textrm{Gd}~4f$ electrons were treated as valence states.

To theoretical study of electronic band structure, we use the tight binding model in the maximally localized Wannier orbitals basis ^62,63^. This model was constructed from exact DFT calculations in a conventional unit cell, with $\textrm{6} \times \textrm{2} \times \textrm{6}$ $\Gamma$-centered **k**–point grid, using the Wannier90 software ^64^. The tight binding ($\textrm{28}$ orbitals $\textrm{112}$ bands) model is based on *d* orbitals of $\textrm{Gd}$, and *p* orbitals of $\textrm{Te}$. Finally, the energy-momentum spectra of the system were calculated using the surface Green's function technique for a semi-infinite system^65^, implemented in WannierTools^66^.

### Supplementary Information

Supplementary Information.
