# Supplementary material for: Observation of momentum-dependent charge density wave gap in a layered antiferromagnet GdTe3
Source: Sci Rep. 2023 Oct 30;13:18618. doi: 10.1038/s41598-023-44851-8 (PMC10616278; doi:10.1038/s41598-023-44851-8)
Supplement: Supplementary file 1 — Supplementary Information. [file 41598_2023_44851_MOESM1_ESM.pdf]

Supplementary Information

Observation of momentum-dependent charge density wave gap in a layered antiferromagnet $GdTe_3$

Sabin Regmi,[1,2] Iftakhar Bin Elius,[1] Anup Pradhan Sakhya,[1] Dylan Jeff,[1,3] Milo Sprague,[1] Mazharul Islam Mondal,[1] Damani Jarrett,[1] Nathan Valadez,[1] Alexis Agosto,[1] Tetiana Romanova,[4] Jiun-Haw Chu,[5] Saiful I. Khondaker,[1,3] Andrzej Ptok,[6] Dariusz Kaczorowski,[4] and Madhab Neupane[1]

[1]*Department of Physics, University of Central Florida, Orlando, Florida 32816, USA*
[2]*Current: Center for Quantum Actinide Science and Technology, Idaho National laboratory, Idaho Falls, Idaho 83415, USA*
[3]*NanoScience Technology Center, University of Central Florida, Orlando, Florida 32826, USA*
[4]*Institute of Low Temperature and Structure Research, Polish Academy of Sciences, Okólna 2, PL-50-422 Wrocław, Poland*
[5]*Department of Physics, University of Washington, Seattle, Washington 98195, USA*
[6]*Institute of Nuclear Physics, Polish Academy of Sciences, W. E. Radzikowskiego 152, PL-31342 Kraków, Poland*

This supplementary material contains supporting information including:

- Heat capacity measurements
- Exfoliation of bulk sample to thin-layered samples and Raman spectroscopy results
- ARPES measured energy contours with 68 eV incident photon energy
- Dispersion maps for 90 eV incident photon energy
- Dispersion map along various angled cuts between $\overline{\Gamma} - \overline{Z}$ and $\overline{\Gamma} - \overline{M}$
- Results of measurement at 15 K

SUPPLEMENTARY NOTE 1: SAMPLE CHARACTERIZATION AND HEAT CAPACITY MEASUREMENTS

High-quality single crystals of $GdTe_3$ were synthesized using the self-flux technique. Chemical composition and phase homogeneity of the crystals were determined by means of energy-dispersive X-ray (EDX) analysis performed using a FEI scanning electron microscope equipped with an EDAX Genesis XM4 spectrometer. The result of EDX analysis is presented in Supplementary Figure 1a, which indicates the desired stoichiometry and homogeneous chemical composition with 26.27 % Gd and 73.73 % Te. Supplementary Figure 1b shows the specific heat (C) of $GdTe_3$ measured up to 400 K, with inset showing a zoomed in view at high temperatures. Despite severe irregularities in the $C(T)$ curve caused by addenda contribution (Apiezon N grease), one can recognize an anomaly near 375 K that arises due to the incommensurate charge density wave formation[1].

SUPPLEMENTARY NOTE 2: RAMAN SPECTROSCOPY AND MECHANICAL EXFOLIATION OF THIN FLAKES

The exfoliation of the thin layer $GdTe_3$ samples was performed via Au-Assisted exfoliation. The metal layer deposition was completed in an electron/thermal evaporation system (Thermionics E-beam & RDM Thermal evaporator). A 2 nm metal adhesion layer of Ti was first evaporated onto a Si/SiO_2 substrate via e-beam evaporation. 5 nm of Au was then evaporated onto the Ti adhesion layer via thermal evaporation. Soon after exposing the Au layer to ambiance, the $GdTe_3$ crystals were cleaved from a parent crystal and lightly pressed onto the target substrate using Nitto tape (SPV-224 PVC) for one minute. Lifting the tape resulted in exfoliated thin layers of $GdTe_3$ on the Au/Ti/SiO_2/Si substrate.

Atomic force microscopy (AFM) was utilized to measure the thickness and identify the layer number of the exfoliated thin layer $GdTe_3$. AFM measurements were taken in non-contact mode using a SmartSPM1000 scanning probe microscope. A single layer of $GdTe_3$ is reported to have a height of 1.2 nm, serving as the baseline for the measurement of the flake's layer number. The AFM measurements were taken in ambient conditions. Optical image of representative thin flakes used in the Raman measurements are presented in Supplementary Figure 2a, where the arrow represents the locatio of where the Raman was measured. The AFM images are presented in Supplementary Figure 2b, with red line indicating the direction which height profile was taken. The height profile of corresponding thin flakes are presented in Supplementary Figure 2c.

In order to ensure that the Raman spectra being reported was of pristine conditions, the bulk $GdTe_3$ was cleaved before any Raman experimentation was done. The Raman spectra for bulk as well as for different layered samples up to 4L thin are presented in Supplementary Figure 2d. In the Raman spectrum, several peaks are observed, which is consistent with those reported in the literature[2,3]. The peaks at 64 cm^{-1} and 79 cm^{-1} represent overlapping degenerate A_g and B_g phonon modes, the peak at 59 cm^{-1} is a two-fold symmetric phonon mode, the peaks at 104 cm^{-1}, 117 cm^{-1}, 129 cm^{-1}, 144 cm^{-1} are B_g symmetric phonons, and the peak at 91 cm^{-1} is an A_g symmetric phonon[3]. Of interest is the peak at 46 cm^{-1}, which has been reported to be the CDW amplitude mode[2,3]. The presence of this strong peak at room temperature Raman data supports the presence of CDW phase at room temperature. In thin films up to 4L, this peak remains strong, indicative of the presence of room-temperature CDW in two-dimensional limit. In fact, it has been documented that the CDW transition shifts to higher temperature with diminishing thickness[2]. Thus, $GdTe_3$ could be an excellent platform for studying how different physical orders such as long-range magnetic, superconducting, charge-density wave, etc. in both three- and two-dimensions.

SUPPLEMENTARY NOTE 3: ARPES MEASURED CONSTANT ENERGY CONTOURS WITH PHOTON ENERGY OF 68 eV

In Supplementary Figure 3, we present the Fermi surface and constant energy contours measured with a photon energy of 68 eV obtained at 8 K. Similar to the observations in the main text Figure 2 (with 90 eV photon energy), the Fermi surface has some intensity along and around the $\overline{\mathrm{X}}-\overline{\Gamma}-\overline{\mathrm{X}}$ direction and no intensity along $\overline{\mathrm{M}}-\overline{\Gamma}-\overline{\mathrm{M}}$ and $\overline{\mathrm{Z}}-\overline{\Gamma}-\overline{\mathrm{Z}}$. Spectral intensity appears at $\sim$ 150 meV binding energy along $\overline{\mathrm{M}}-\overline{\Gamma}-\overline{\mathrm{M}}$ and about $\sim$ 320 meV binding energy along $\overline{\mathrm{Z}}-\overline{\Gamma}-\overline{\mathrm{Z}}$.

SUPPLEMENTARY NOTE 4: OBSERVATION OF CDW BANDS

In Supplementary Figure 4a, we present an integrated energy contour taken with 68 eV incident photon energy. The integration is done within a 50 meV window centered at the binding energy of 300 meV. Similar to the observation for 90 eV photon energy presented in the main text Figure 2h, the features that can not be described by the non-CDW calculations appear (shown by magenta colored arrow in 4a and represented by magneta colored dashed curves in 4b). An energy-momentum dispersion cut taken along the yellow colored dashed line in 4b at $k_x = -0.5$ Å^{-1} is presented in Supplemental Figure 4c, where the intense main bands and shadow bands coming from both the band folding and CDW can be observed.

SUPPLEMENTARY NOTE 5: $\overline{\mathrm{M}}-\overline{\Gamma}-\overline{\mathrm{M}}$ AND $\overline{\mathrm{Z}}-\overline{\Gamma}-\overline{\mathrm{Z}}$ DISPERSION MAPS (90 eV)

In Supplementary Figure 5, we present the dispersion maps taken along the $\overline{\mathrm{M}}-\overline{\Gamma}-\overline{\mathrm{M}}$ and $\overline{\mathrm{Z}}-\overline{\Gamma}-\overline{\mathrm{Z}}$ directions taken from measurements using 90 eV photon energy. From the dispersion maps (5a and 5d) and their second derivative plots (5b and 5e), it is clear that the gap size below the Fermi level for 90 eV data are around 140 meV and 310 meV along $\overline{\mathrm{M}}-\overline{\Gamma}-\overline{\mathrm{M}}$ and $\overline{\mathrm{Z}}-\overline{\Gamma}-\overline{\mathrm{Z}}$, respectively in agreement with the observations in the constant energy contours presented in the main text Figure 2. In Supplementary Figures 5c and 5f, we present the theoretically calculated surface spectrum along the $\overline{\mathrm{M}}-\overline{\Gamma}-\overline{\mathrm{M}}$ and $\overline{\mathrm{Z}}-\overline{\Gamma}-\overline{\mathrm{Z}}$ directions, respectively. While the spectrum overall matches the experimental observation, the gap is not seen in the calculations as they are carried out for the non-CDW case.

SUPPLEMENTARY NOTE 6: DISPERSION MAPS ALONG $\overline{\mathrm{X}}-\overline{\Gamma}-\overline{\mathrm{X}}$ AND $\overline{\mathrm{M}}-\overline{\mathrm{X}}-\overline{\mathrm{M}}$

Supplementary Figure 6(a) represents the dispersion map along the $\overline{\Gamma}-\overline{\mathrm{X}}$ direction obtained from the measurement using a photon energy of 90 eV. Bands crossing the Fermi level can be clearly observed, indicative of the absence of gap along this direction. In Supplementary Figure 6(c), we show the dispersion map along the $\overline{\mathrm{M}}-\overline{\mathrm{X}}$ direction. Around the $\overline{\mathrm{X}}$ point, bands are crossing the Fermi level, which again indicated the absence of any gap around the $\overline{\mathrm{X}}$ point as observed in the Fermi surface maps. The experimentally observed band dispersions along $\overline{\Gamma}-\overline{\mathrm{X}}$ and $\overline{\mathrm{M}}-\overline{\mathrm{X}}$ are well reproduced in the theoretical surface spectrum calculated without considering CDW ordering [Figures 6b,d].

SUPPLEMENTARY NOTE 7: DIRECTION DEPENDENCE OF THE CDW GAP

In Supplementary Figure 7, we present the dispersion maps, integrated EDCs and the Fermi fits of the leading edge for cut directions $\overline{\Gamma}-\widetilde{\mathrm{A}}$ through $\overline{\Gamma}-\widetilde{\mathrm{D}}$ as defined in Main text Figure 4. A clear signature of gap decreasing as the direction moves from $\overline{\Gamma}-\widetilde{\mathrm{A}}$ towards $\overline{\Gamma}-\widetilde{\mathrm{D}}$ can be observed. The results of the measurement taken at a temperature of 15 K do not show significant differences (see Supplementary Figure 8).

Supplementary References

[1] Yumigeta, K. et al. Advances in Rare-Earth Tritelluride Quantum Materials: Structure, Properties, and Synthesis. *Adv. Sci.* **8,** 2004762 (2021).
[2] Chen, Y. et al. Raman spectra and dimensional effect on the charge density wave transition in $GdTe_3$. *Appl. Phys. Lett.* **115,** 151905 (2019).
[3] Wang, Y. et al. Axial Higgs mode detected by quantum pathway interference in $R$$Te_3$. Nature **606,** 896 (2022).

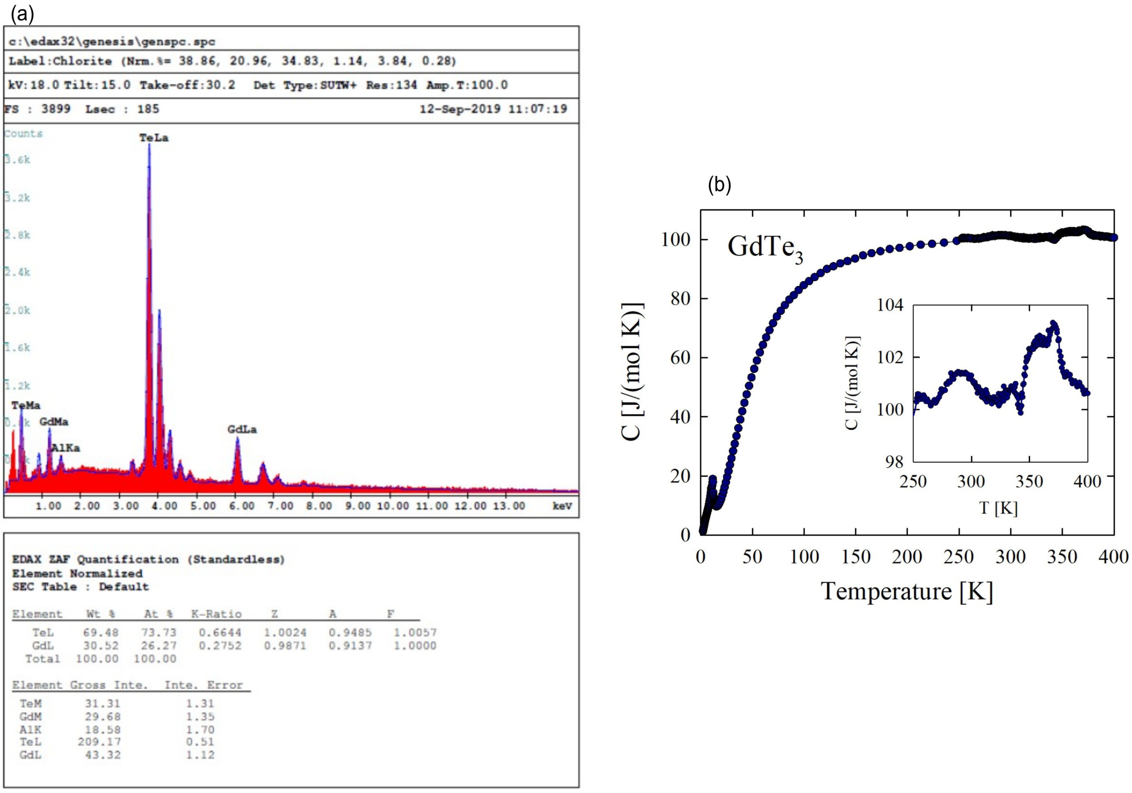

Supplementary Figure 1: **a** Microprobe analysis of the chemical composition of $GdTe_3$ single crystal. **b** Temperature variation of the specific heat over a wide range of temperature. Inset: the data collected in the vicinity of the CDW transition.

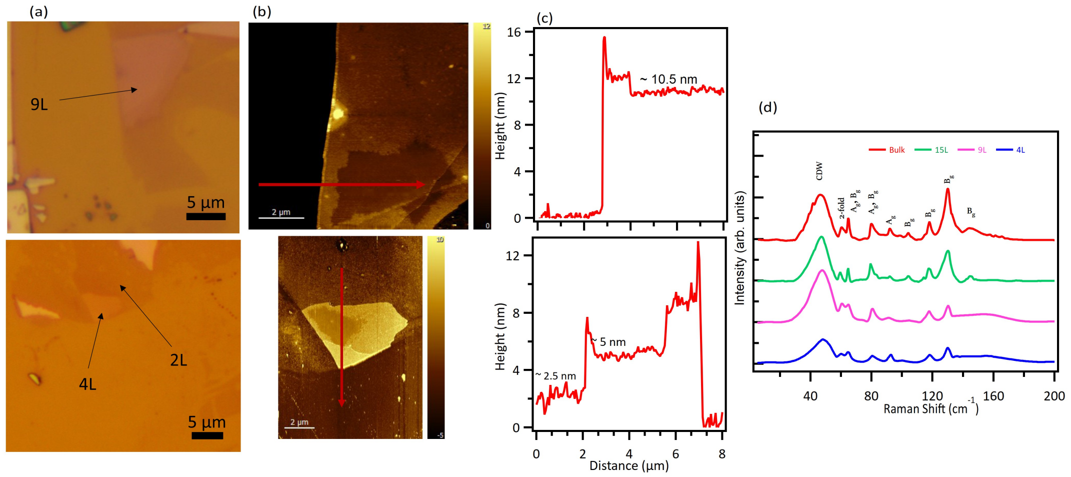

Supplementary Figure 2: Mechanical exfoliation of $GdTe_3$ flakes and Raman spectroscopy results. **a** Optical image, **b** AFM image, and **c** height profiles of $GdTe_3$ flakes exfoliated from bulk crystals. **d** Raman spectroscopy results on bulk and few layered samples indicated.

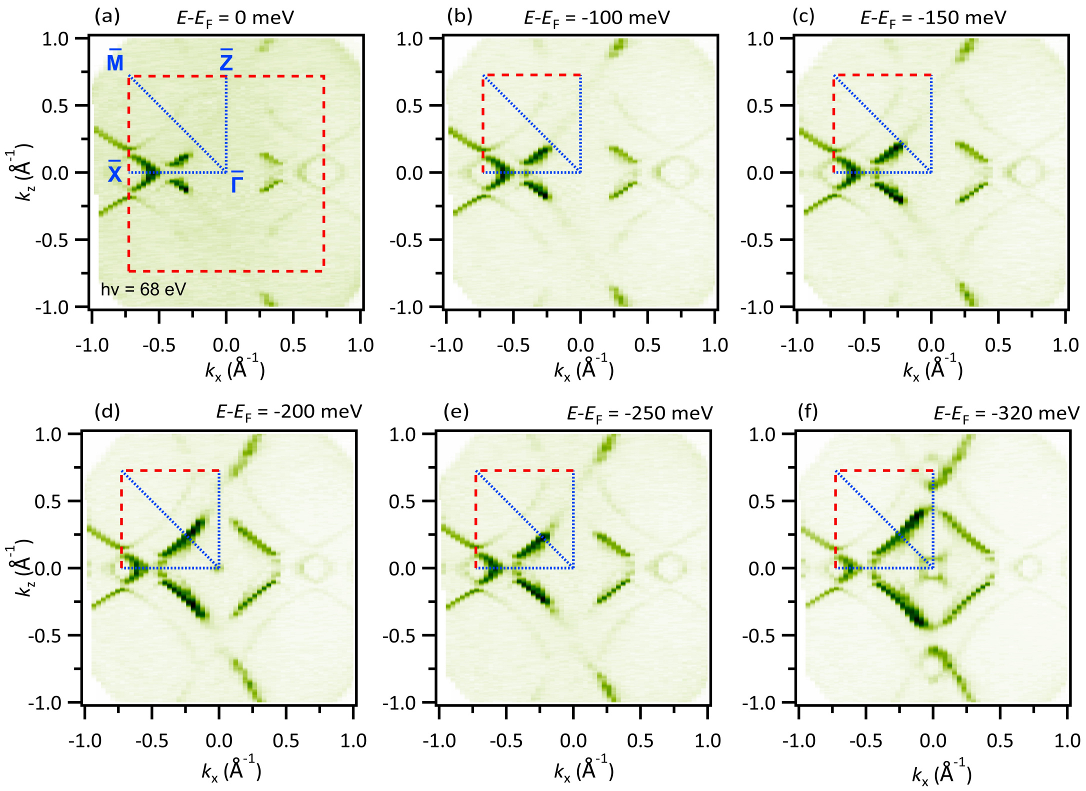

Supplementary Figure 3: Constant energy contours obtained for 68 eV photon energy at binding energies of **a** 0 meV (FS), **b** 100 meV , **c** 150 meV , **d** 200 meV , **e** 250 meV , and **f** 320 meV (experimental Brillouin zone in green and high-symmetry lines/points in cyan color). Data were collected at the SSRL beamline 5-2 at a temperature of 8 K .

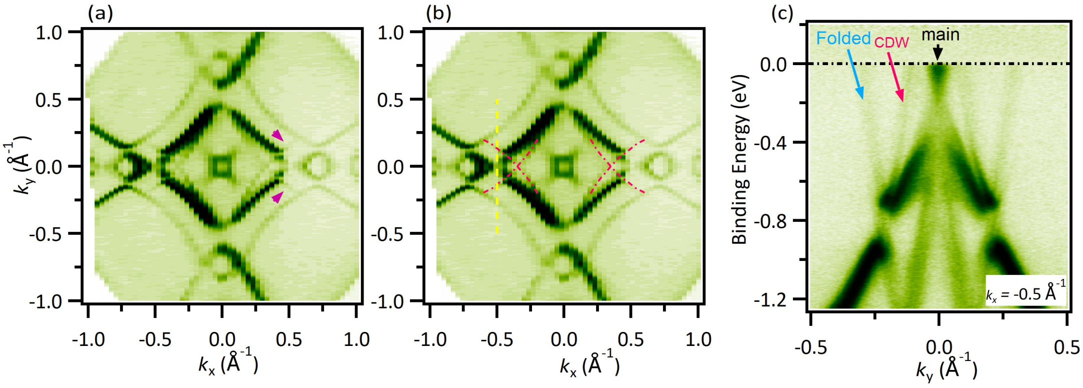

Supplementary Figure 4: Observation of CDW bands. **a** Energy contour integrated within a 50 meV window centered at 300 meV binding energy. **b** Same energy contour as in (a) with CDW induced features represented by magenta colored dashed curves. **c** Dispersion map taken for a $k_x = 0$ $Å^{-1}$ cut represented by the yellow dashed line in (b).

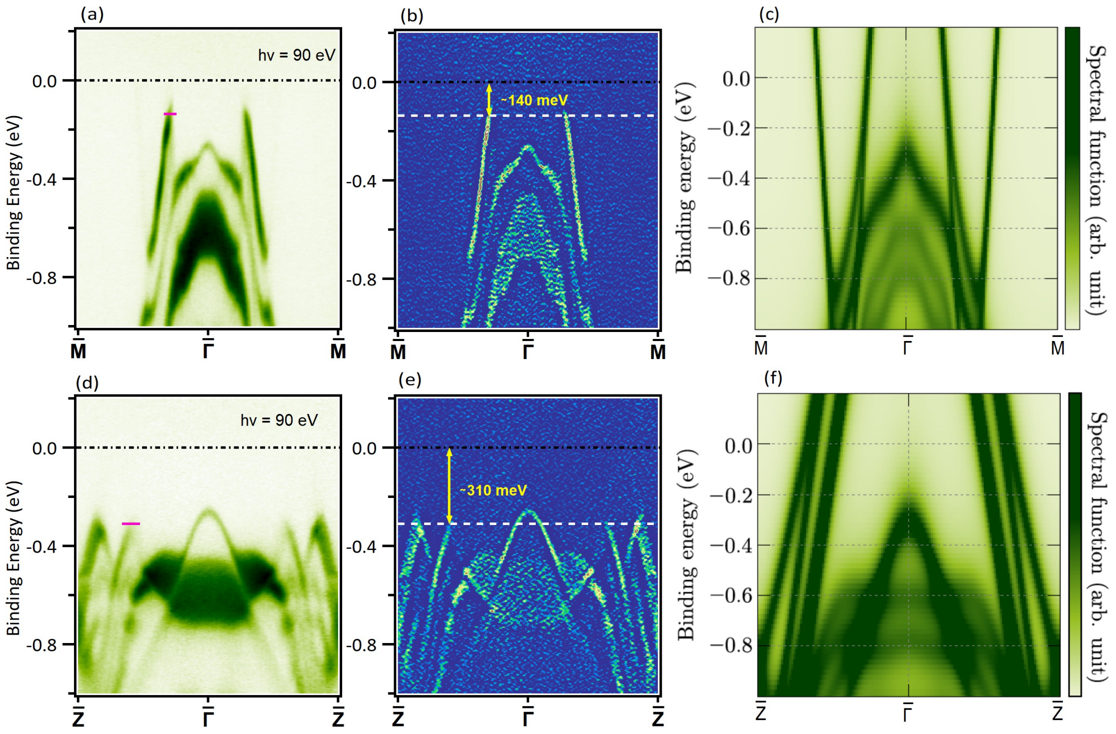

Supplementary Figure 5: Dispersion maps for 90 eV measurements. **a** Dispersion map and **b** its second derivative along $\overline{\mathrm{M}}-\overline{\Gamma}-\overline{\mathrm{M}}$. **c** Calculated surface spectrum along $\overline{\mathrm{M}}-\overline{\Gamma}-\overline{\mathrm{M}}$ without taking into account the CDW ordering. **d** Dispersion map and **e** its second derivative along $\overline{\mathrm{Z}}-\overline{\Gamma}-\overline{\mathrm{Z}}$. **f** Calculated surface spectrum along $\overline{\mathrm{Z}}-\overline{\Gamma}-\overline{\mathrm{Z}}$ without considering CDW order.

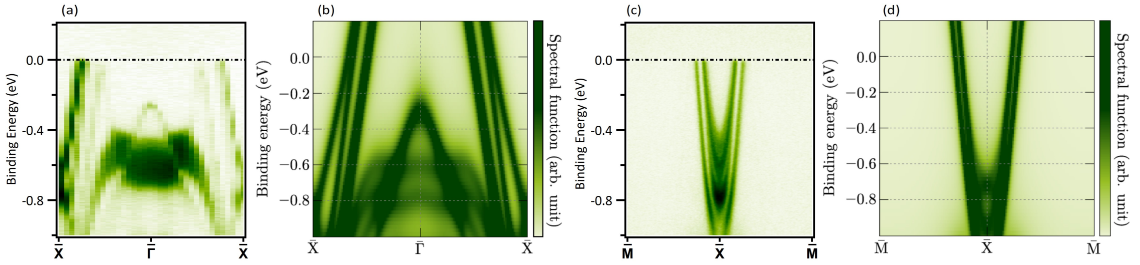

Supplementary Figure 6: Dispersion maps along $\overline{\mathrm{X}}-\overline{\Gamma}-\overline{\mathrm{X}}$ and $\overline{\mathrm{M}}-\overline{\mathrm{X}}-\overline{\mathrm{M}}$. **a** Dispersion map along $\overline{\mathrm{X}}-\overline{\Gamma}-\overline{\mathrm{X}}$. **b** Calculated surface spectrum ($\overline{\mathrm{X}}-\overline{\Gamma}-\overline{\mathrm{X}}$) projection on the (010) surface. **c** Dispersion map along $\overline{\mathrm{M}}-\overline{\mathrm{X}}-\overline{\mathrm{M}}$. **d** Calculated surface spectrum along $\overline{\mathrm{M}}-\overline{\mathrm{X}}-\overline{\mathrm{M}}$ projected on the (010) surface.

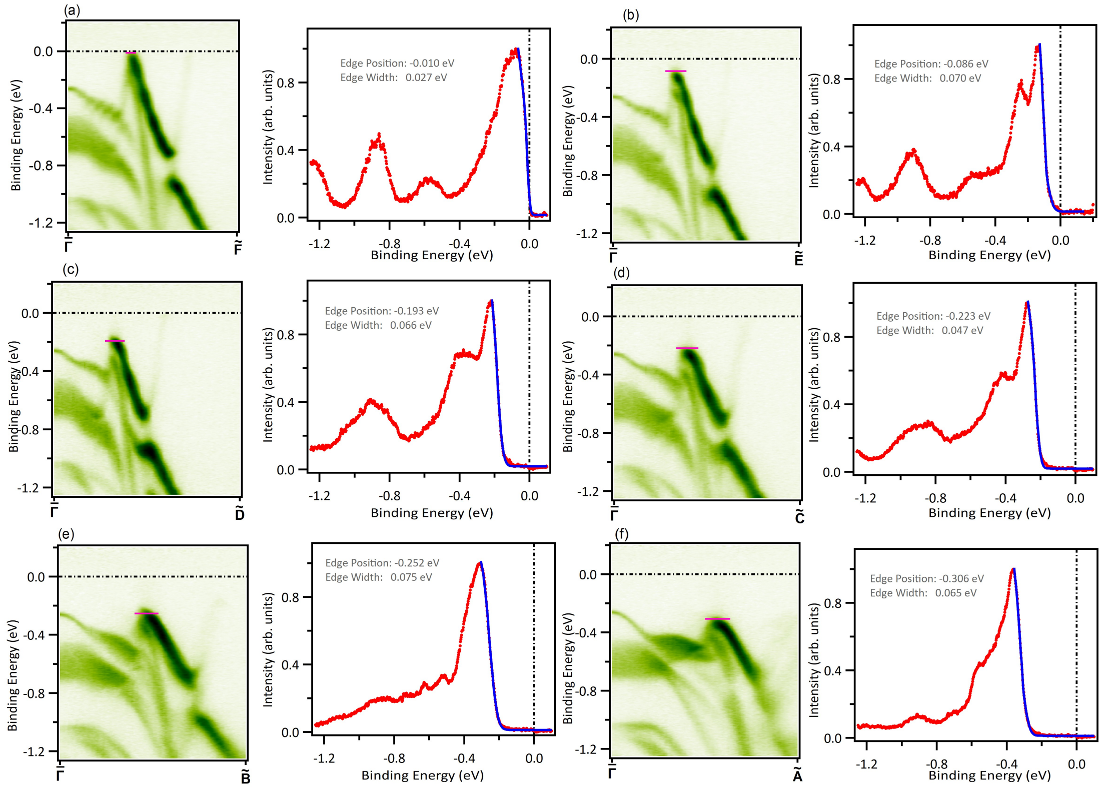

Supplementary Figure 7: Direction dependent gap. Dispersion maps (left panel) and the energy distribution curves integrated within the momentum window indicated by the magenta lines in the dispersion maps (right panel) along **a** $\overline{\Gamma}-\widetilde{\mathrm{F}}$, **b** $\overline{\Gamma}-\widetilde{\mathrm{E}}$, **c** $\overline{\Gamma}-\widetilde{\mathrm{D}}$, and **d** $\overline{\Gamma}-\widetilde{\mathrm{C}}$, **e** $\overline{\Gamma}-\widetilde{\mathrm{B}}$, **f** $\overline{\Gamma}-\widetilde{\mathrm{A}}$. These directions represent the cuts at an angle of respectively: 62°, 55°, 40°, 30°, 20°, and 10°, from the $\overline{\Gamma}-\overline{\mathrm{Z}}$ direction. The leading edges have been fitted by Fermi edge fitting.

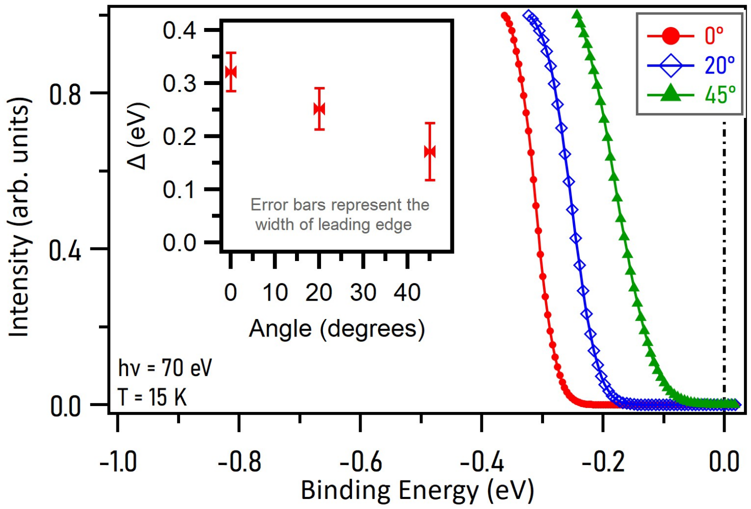

Supplementary Figure 8: Fitted leading edges (normalized) for the angles noted in the plot. Inset: Gap size below the Fermi level, where the error bars represent the width of the leading edge. Data were taken at the ALS beamline 10.0.1.2 at a temperature of 15 K.
